# A miniaturized mouse extracorporeal circulation model to characterize early hematologic and metabolic alterations

**DOI:** 10.3389/fphys.2026.1740282

**Published:** 2026-02-13

**Authors:** Xiaoyu Wang, Xiaohan Cheng, Xinzhi Pan, Yijie Guo, Ruishi Shen, Yueyang Zhu, Adili Abudourousuli, Jiahao Guo, Qi Peng, Huifang Tang, Huashun Cui

**Affiliations:** 1 Shuguang Hospital, Shanghai University of Traditional Chinese Medicine, Shanghai, China; 2 School of Pharmaceutical Sciences, Zhejiang Chinese Medical University, Hangzhou, China; 3 Animal Experimental Center, Xinjiang Medical University, Urumqi, China; 4 Department of Pharmacology, Zhejiang University School of Medicine, Hangzhou, China

**Keywords:** animal models, cardiovascular system, extracorporeal circulation, mouse, pathophysiology

## Abstract

**Introduction:**

Extracorporeal circulation (ECC) is essential in cardiac surgery but triggers severe complications like systemic inflammation, coagulopathy, and end-organ damage. Progress in understanding these events has been hampered by the high cost and complexity of medium-animal models, alongside the technical challenges of operating on mice.

**Methods:**

To address this, we developed a miniaturized, pre-configured murine ECC system that reliably recapitulates key clinical sequelae of ECC. This novel model, established in C57BL/6 mice, utilizes a low-cost, single-use circuit to maximize reproducibility and minimize contamination. Throughout the procedure, key physiological parameters were monitored and maintained stable.

**Results:**

Our system successfully induced hallmark acute-phase responses to ECC, including a systemic inflammatory response (leukocytosis and elevated pro-inflammatory cytokines including IL-1β, IL-6, TNF-α, and IL-18), consumptive coagulopathy (thrombocytopenia), and metabolic stress (elevated lactate levels and electrolyte disturbances). Additionally, early biomarkers associated with organ stress were detected, including elevated cardiac troponin and LDH, increased serum creatinine, elevated AST, and increased pulmonary myeloperoxidase activity.

**Discussion:**

With stable core temperature and pH underscoring the system’s controllability, this model provides a reproducible experimental platform for investigating the early molecular mechanisms of ECC-induced systemic responses and for supporting preclinical evaluation of potential therapeutic interventions in cardiovascular research.

## 1 Introduction

Extracorporeal circulation (ECC) is a fundamental technology in open-heart surgery, markedly advancing modern cardiac surgery and proving instrumental in saving countless patients (Li et al., 2025). However, ECC triggers a systemic inflammatory response that frequently results in severe complications, such as neurocognitive dysfunction, pulmonary injury, acute kidney injury, and multiple organ failure (Jones et al., 2019; Sun et al., 2021). These adverse outcomes substantially diminish postoperative quality of life and place a considerable burden on healthcare systems. The precise molecular mechanisms driving ECC-induced organ damage remain inadequately understood, underscoring the need for the development of reliable animal models to investigate the associated pathophysiological processes. In this study, “ECC” refers to a short-term, normothermic, partial veno-arterial extracorporeal circulation model operated in parallel with an intact native heart, and is therefore distinct from conventional cardiopulmonary bypass (CPB), which replaces native cardiac function intraoperatively, as well as from extracorporeal membrane oxygenation (ECMO), which is designed for prolonged life support.

Currently, commonly employed medium-size animal models, such as rats and rabbits, exhibit several limitations for ECC research, including complex management, substantial blood volume requirements, and challenges in physiological monitoring (Rahman et al., 2023). More importantly, they also lack a wide range of genetically modified strains, which restricts comprehensive mechanistic studies. In contrast, murine models offer distinct advantages for the detailed investigation of ECC-related genes and signaling pathways, owing to their extensive genetic toolkits, small size, cost-effectiveness, and well-characterized genetic backgrounds (Rosenthal and Brown, 2007).

Consequently, the establishment and optimization of murine ECC models are crucial to significantly enhance our understanding of the ECC induced systemic inflammatory mechanisms, and to provide essential technical foundations for the development of targeted therapeutic interventions.

## 2 Materials and methods

### 2.1 Mouse strains

C57BL/6 mice (25–30 g, male) were purchased from Shanghai SLAC Laboratory Animal Co., Ltd. (Shanghai, China; production license SCXK [Hu] 2022-0004). The mice were housed under specific-pathogen-free (SPF) conditions with a 12 h light/12 h dark cycle, and had *ad libitum* access to irradiated standard rodent chow and autoclaved tap water. All experimental procedures were approved by the Animal Ethics Committee of Zhejiang University (approval No. ZJU20240505) and were conducted in accordance with the international Guidelines for the Care and Use of Laboratory Animals.

Eighteen male C57BL/6 mice (25–30 g) were used in this study and randomly divided into three groups (n = 6 per group): the Control group, which received no procedural intervention; the Sham group, which underwent surgical exposure of the jugular vein and femoral artery without cannulation, to account for the effects of anesthesia and surgical trauma; in brief, mice in the Sham group received the same preoperative preparation as the ECC group, including anesthesia and surgical dissection/exposure of the jugular vein and femoral artery. No vascular cannulation was performed and the extracorporeal circuit was not connected or initiated and the ECC group, in which the exposed vessels were cannulated to conduct ECC as the experimental model under investigation.

### 2.2 Murine miniaturized extracorporeal circulation circuit

The murine extracorporeal circulation (ECC) system was designed as a miniaturized, closed-loop veno-arterial circuit providing partial circulatory support, operating in parallel with an intact, beating native heart rather than replacing total cardiac output. This configuration was intended to model partial extracorporeal circulatory assistance rather than full cardiopulmonary bypass. The circuit is primarily composed of the following components, connected in series: The circuit sequence was: right external jugular vein (drainage) → pump → membrane oxygenator → blood storage room (mini-reservoir) → left femoral artery (return). A miniature peristaltic pump (YZ1515, Baoding Qili Constant Flow Pump Co., Ltd., China) served as the artificial heart, providing the motive force for blood circulation. The pump was driven by its corresponding drive unit (BT100-02, Baoding Qili Constant Flow Pump Co., Ltd., China), which allowed for precise digital control of the flow rate. According to the manufacturer’s specifications, the pump operates under a wide flow range (0.07–2,400 mL/min, ≤600 rpm), enabling accurate regulation of the low-flow conditions required for murine ECC. The pump head was fitted with a segment of pharmaceutical-grade pump tubing (Pharmed 13#, Baoding Qili Constant Flow Pump Co., Ltd., China), which featured a proprietary anticoagulant coating on its inner surface to enhance hemocompatibility. The outlet of the pump tubing was connected to a miniature membrane oxygenator (Micro-MO, Dongguan Kewei Medical Device Co., Ltd., China) for gas exchange, effectively functioning as an artificial lung. According to the manufacturer, the oxygenator consists of a polycarbonate housing with polypropylene hollow-fiber membranes and provides a total membrane surface area of approximately 0.05 m^2^. The oxygenator demonstrated stable gas exchange performance within the experimental flow range applied in this study (2.5–3.5 mL/min/100 g body weight). The oxygenated blood was then passed through a standard disposable medical three-way stopcock and a positive-pressure sterile infusion set (Guangdong Baihe Medical Technology Co., Ltd., NMPA Registration No. 20223140499) for fluid administration and sample collection. For vascular access, a 26G intravenous catheter (Shanghai Kindly Medical Group, NMPA Approval No. 20193141674 Co., Ltd., China) was used for cannulation of the right external jugular vein as the drainage (venous) line. A component labeled as “blood storage room” was incorporated into the circuit upstream of the arterial return line. It was assembled using a sterile micro 3-way Y connector (22G) (WP32001, Instech Laboratories, Inc., United States) connected to a modified 1-mL Luer-Lok™ syringe barrel (BD 309628, Becton, Dickinson and Company (BD), United States), forming a closed mini-reservoir. The left femoral artery was similarly cannulated using a 26G intravenous catheter of the same specification (Shanghai Kindly Medical Group, NMPA Approval No. 20193141674 Co., Ltd., China), which served as the return (arterial) line. The circuit was interconnected using 22G PE/PVC tubing (btcoex-22, Beijing Mingtai Jiaxin Technology Co., Ltd., China) to complete the closed-loop path. All components were meticulously assembled and primed prior to the procedure to ensure a sterile, bubble-free, and closed system.

### 2.3 Surgery

#### 2.3.1 ECC preparation

**FIGURE 1:**
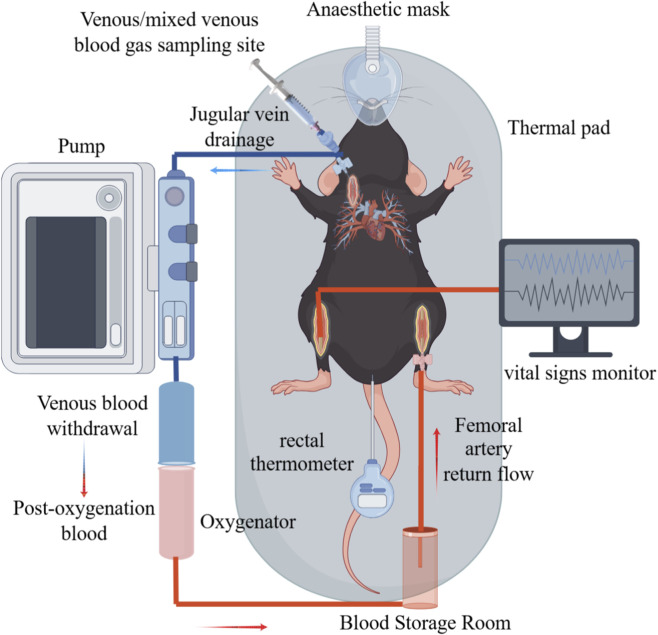
Miniaturized extracorporeal circulation circuit. The illustration depicts a mouse under inhalation anesthesia, instrumented with venous cannulas for infusion/sampling and a rectal probe for temperature monitoring. The system includes an oxygenator, a circulatory pump, and real-time vital sign monitoring, demonstrating a comprehensive life support and monitoring system.

All surgical procedures were performed under sterile conditions within a specific-pathogen-free (SPF) animal facility, using autoclaved surgical instruments and sterile disposable materials. Given the acute nature of the experiment (fixed ECC duration of 60 min), no long-term survival or postoperative recovery was required. Nevertheless, standard aseptic techniques were strictly applied throughout the procedure, including preoperative site preparation and the use of sterilized instruments, to minimize contamination-related confounding. Under these conditions, the surgical protocol is appropriate for short-term intravascular cannulation models and ensures experimental reproducibility without introducing infection-related bias. The core ECC system configuration is shown in Figure 1. Prior to the experiment, the circuit was primed and circulated for 30 min at a flow rate of 0.5 mL/min using a 1:1 mixture of homologous mouse plasma and heparinized compound electrolyte solution, containing sodium heparin (200 IU/kg), delivered by a peristaltic pump, ensuring uniform activation of the anticoagulant coating. The use of a plasma-containing priming solution was intended to provide endogenous plasma proteins for surface conditioning, which has been shown to improve hemocompatibility and attenuate complement and platelet activation during initial blood–artificial surface contact in extracorporeal circuits (Cho et al., 2021). In addition, the 1:1 mixing ratio was selected to preserve physiological colloid osmotic pressure while maintaining adequate circuit priming and anticoagulation (Kayumov et al., 2023). Based on preliminary pilot experiments conducted prior to the formal study, the total priming volume was limited to 0.8–1.0 mL to minimize hemodilution and dilution-related physiological confounding, consistent with the design principles of miniaturized ECC systems.

#### 2.3.2 Conduct of ECC

Mice were anesthetized via induction with 5% sevoflurane to maintain spontaneous and regular breathing (Voiriot et al., 2017). To counteract heat loss, the anesthetized mice were secured on a thermostatically controlled heating pad maintained at a constant 37 °C throughout the preparatory phase. Following anesthetic induction, surgical anesthesia was maintained with 2% sevoflurane delivered via a nose cone (Song et al., 2025). Anesthetic depth was assessed by monitoring the pedal withdrawal reflex (hind paw pinch), which was evaluated at regular intervals during the procedure to ensure an adequate and stable depth of anesthesia. During ECC, animals were maintained under assisted/controlled ventilation rather than spontaneous breathing to ensure stable respiratory conditions and consistent gas exchange throughout the experiment. The mouse was positioned supine on a thermostatically controlled heating pad, which was maintained at 37 °C throughout the procedure. Surgical interventions were performed under strict aseptic conditions using autoclaved instruments to minimize contamination. Specifically, following shaving and disinfection of the surgical sites, new sterile gloves were donned immediately prior to the cannulation procedure. Given the acute nature of the experiment (60-min duration), this standard aseptic protocol was sufficient to maintain physiological stability and prevent infection-related confounding factors.

All procedures followed a standardized and identical protocol across animals, and all surgeries were performed by the same investigator to minimize operator-related variability. The total surgical time was approximately 20–25 min per animal (from skin incision to ECC initiation), with no meaningful inter-animal variation. ECC was maintained for 60 min in all animals in the ECC group.

#### 2.3.3 Surgical cannulation

For jugular venous access, the mouse’s head was gently extended. A superficial incision was made to expose the right external jugular vein, which was identified as a dark red vessel approximately 0.3–0.5 mm in diameter, located 3–5 mm superior to the clavicle-sternum junction and lateral to the sternocleidomastoid muscle (Sen et al., 2015). The optimal site for cannulation was selected at the junction of the transverse jugular and suprascapular veins, approximately 2–3 mm above the clavicle, where the vessel diameter is maximal and the risk of bleeding is minimal. The vein was meticulously dissected free from the surrounding connective tissue using micro-forceps in preparation for ligation and cannulation.

For femoral arterial access, the left hindlimb was abducted and externally rotated. A 5 mm longitudinal incision was made 2–3 mm distal to the inguinal groove, directly over the palpable arterial pulse (Gaetano et al., 2013). The femoral artery, approximately 0.2–0.3 mm in diameter and bright red, was exposed through blunt dissection between the pectineus and adductor muscles, ensuring that the closely associated femoral vein (medially) and femoral nerve (laterally) were not injured. The superficial epigastric artery, which branches distally, served as a crucial anatomical landmark. The artery was adequately mobilized to facilitate secure ligation and cannulation.

Systemic heparinization was achieved by administering 200 IU/kg of sodium heparin via the tail vein. Distal ligatures were placed on both vessels using micro-forceps. The venous cannula was advanced toward the right atrium, followed by the insertion of the arterial cannula, which was directed retrogradely toward the aortic bifurcation. Once both cannulae were secured, ECC was initiated, establishing a closed-loop circuit from the jugular vein for drainage to the femoral artery for reinfusion. This cannulation configuration (right external jugular vein for venous drainage and left femoral artery for arterial return) was identical for all animals in the ECC group.

Of note, aortic cross-clamping was not performed in this model. The heart remained intact and spontaneously beating throughout the ECC procedure, allowing isolation of ECC-specific systemic inflammatory and coagulopathic effects without the confounding influence of myocardial ischemia–reperfusion injury.

Following confirmation of circuit patency, ECC flow was gradually initiated. The peristaltic pump flow rate was incrementally increased to 2.5–3.5 mL/min/100 g body weight, which was selected to provide partial veno-arterial extracorporeal support in parallel with an intact, spontaneously beating heart (aortic cross-clamping was not performed). This target range was physiologically defined based on expected cardiac output under volatile anesthesia and the intended degree of partial support. Specifically, resting cardiac output in conscious adult mice is ∼6–8 mL/min/100 g (Janssen et al., 2002), whereas clinically relevant concentrations of sevoflurane reduce cardiac output by ∼15%–30% (Hanouz et al., 2000; Roth et al., 2002; Gentry-Smetana et al., 2008), yielding an estimated baseline of ∼4–5 mL/min/100 g under our conditions. Therefore, an ECC flow of 2.5–3.5 mL/min/100 g was chosen to deliver approximately half to two-thirds of anesthetized baseline cardiac output while avoiding excessive venous drainage or arterial afterload. The upper limit (3.5 mL/min/100 g) was set as a conservative safety threshold to minimize microcirculatory and inflammatory perturbations associated with small-animal ECC (Ki et al., 2020; Cho et al., 2021), whereas the lower limit (2.5 mL/min/100 g) was selected to maintain adequate basal oxygen delivery under anesthesia with an added margin for circuit inefficiency (Pacher et al., 2008). Flow escalation was performed gradually to prevent acute hemodynamic compromise, including hypotension from excessive venous drainage or pressure overload from aggressive arterial reinfusion (Austin et al., 2020).

As blood flowed through the miniature membrane oxygenator, efficient gas exchange was sustained, effectively simulating pulmonary function by oxygenating the blood and eliminating carbon dioxide. The circuit underwent continuous inspection to ensure a bubble-free, uninterrupted blood column from venous outflow to arterial return, thereby confirming system integrity. Core body temperature was maintained at 37.0 ± 0.5° Cusing a heating pad, while vital signs, including heart rate and respiratory rate, were monitored continuously. Continuous arterial blood pressure and direct cardiac output measurements were not recorded in the present study, and this limitation is acknowledged. Arterial blood gas analysis was conducted at baseline 0 min, 30 min, and 60 min after the initiation of ECC to quantitatively assess physiological status and the efficacy of gas exchange. At the end of the 60-min ECC period, mice were euthanized under deep sevoflurane anesthesia by cervical dislocation, in accordance with the institutionally approved animal protocol and internationally accepted humane euthanasia guidance for laboratory rodents. Death was confirmed by cessation of respiration and cardiac activity prior to final tissue harvest.

### 2.4 Blood gas analysis

**FIGURE 2:**
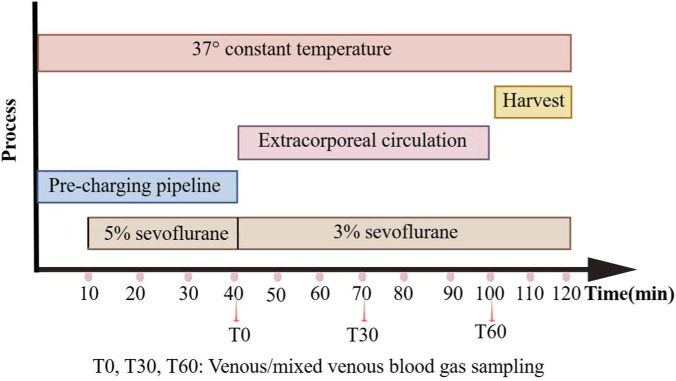
Modeling process and timeline for blood gas and blood cell testing. Colored horizontal bars represent the duration of each procedural step: pipeline pre-charging, anesthesia maintenance (5% and 3% sevoflurane), extracorporeal circulation, and final tissue harvest, all maintained at 37 °C.

Circuit-Derived mixed venous blood sample (approximately 100 μL each) for evaluating systemic oxygenation, ventilation, and metabolic status during ECC were collected at three specified time points: baseline (BL), post anesthetic stabilization, 30 min post ECC initiation (T30), and 60 min post ECC initiation (T60) (Figure 2). The samples were obtained using heparinized syringes connected to the venous line through a three-way stopcock, ensuring the immediate removal of any air bubbles upon collection. Analysis of all samples was conducted within 60 s of collection using a Radiometer ABL90 FLEX blood gas analyzer (Radiometer, Denmark), which underwent daily automatic calibration. The parameters measured included pH, bicarbonate (HCO_3_^−^), base excess (BE), lactate, and potassium (K^+^), with all values reported at 37 °C. It should be emphasized that these measurements reflect mixed venous (pre-oxygenator) blood gas and electrolyte composition rather than true systemic arterial values, and are therefore interpreted as indicators of global metabolic status rather than arterial oxygenation performance.

### 2.5 Blood cell analysis

To evaluate the dynamic changes in blood composition during ECC, additional blood samples were collected in heparin-anticoagulated tubes at the same time points as the blood gas analyzes: baseline, 30 min on ECC, and 60 min on ECC. Following blood gas measurement, the remaining sample in the heparinized syringe was gently inverted several times to prevent clotting. A 20 μL aliquot was then aspirated and analyzed using a URIT-290 Vet Plus automated hematology analyzer (URIT Medical Electronic, China) to determine the counts of white blood cells (WBC), red blood cells (RBC), and platelets (PLT) at each stage. Daily quality control procedures were conducted using manufacturer-matched calibrators to ensure the accuracy and reliability of all measurements.

The above procedure outlines the perfusion circuit, surgical procedures, and monitoring of physiological parameters during ECC in a murine model. When performed by a microsurgeon with adequate expertise, the results are consistently reproducible.

### 2.6 Enzyme-linked immunosorbent assay (ELISA)

Plasma samples were collected from mice, and the levels of pro-inflammatory cytokines (IL-1β, IL-6, TNF-α, and IL-18) were quantified using commercially available enzyme-linked immunosorbent assay (ELISA) kits according to the manufacturers’ instructions. Briefly, whole blood was collected into anticoagulant-treated tubes and centrifuged at 1,500–2,000 × g for 10 min at 4 °C to obtain plasma. The plasma samples were aliquoted and stored at −80 °C until analysis. ELISA kits were obtained as follows: IL-1β (Cat. No. 88-7013a, Invitrogen, Thermo Fisher Scientific, United States), IL-6 (Cat. No. DY406, R&D Systems, United States), TNF-α (Cat. No. 88-7324, Invitrogen, Thermo Fisher Scientific, United States), and IL-18 (Cat. No. SEK50073, Sino Biological Inc., China). All samples were analyzed strictly according to the manufacturers’ instructions.

### 2.7 Measurement of organ injury–related biomarkers

Organ injury–related biomarkers in mouse plasma were measured using commercially available assay kits according to the manufacturers’ instructions, with plasma samples prepared as described above. Creatinine (Cr) was measured to assess renal injury (Cat. No. C011-2-1, Nanjing Jiancheng Bioengineering Institute, China); cardiac troponin and lactate dehydrogenase (LDH) were used to evaluate cardiac injury (Cat. No. EM30867S, Weiao Biotechnology, China; Cat. No. A020-4-1, Nanjing Jiancheng Bioengineering Institute, China); aspartate aminotransferase (AST/GOT) was measured as a marker of hepatic injury (Cat. No. C010-2-1, Nanjing Jiancheng Bioengineering Institute, China); and myeloperoxidase (MPO) activity was determined to assess pulmonary injury (Cat. No. A044-1-1, Nanjing Jiancheng Bioengineering Institute, China). All samples were analyzed strictly according to the manufacturers’ instructions.

### 2.8 Statistical analyzes

Statistical analyzes were performed using GraphPad Prism software (version 10.1.2; GraphPad Software, San Diego, CA, United States). All data are presented as mean ± standard error of the mean (SEM). Prior to parametric testing, data normality was assessed using the Shapiro–Wilk test, and homogeneity of variance was evaluated using Levene’s test (or Brown–Forsythe test, where appropriate). All datasets satisfied the assumptions required for analysis of variance. For comparisons among the three experimental groups (Control, Sham, and ECC) at the final time point (60 min), a one-way analysis of variance (ANOVA) was performed, followed by Tukey’s *post hoc* test for multiple comparisons. For comparisons across time within the ECC group (0, 30, and 60 min), a one-way repeated-measures ANOVA was applied. Sphericity was assessed using Mauchly’s test, and when violations were detected, the Greenhouse–Geisser correction was applied automatically. No data points were excluded from the final analyzes. All measurements were successfully obtained at all predefined time points, therefore, no missing data imputation was required.

## 3 Results

### 3.1 ECC-driven leukocytosis and lymphopenia

**FIGURE 3:**
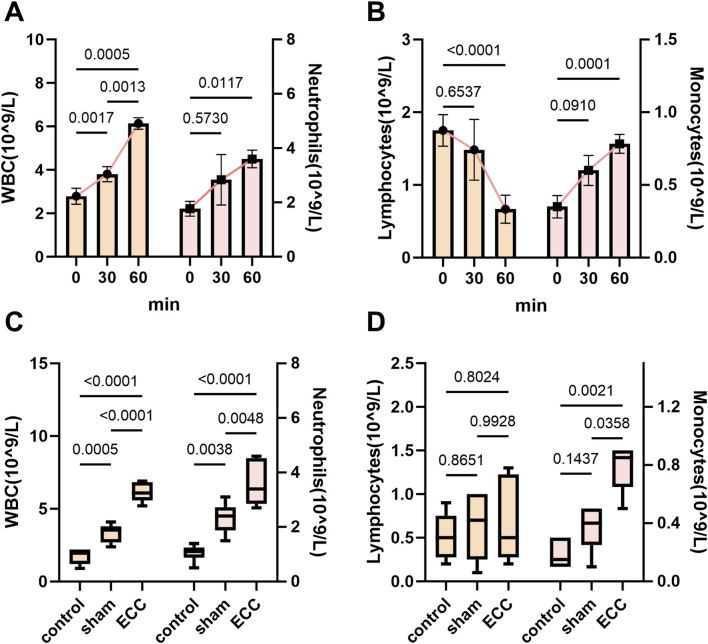
Neutrophil-driven leukocytosis and lymphopenia during early ECC. **(A)** Time course of WBC (left y-axis) and neutrophils (right y-axis) measured at baseline (0 min) and during ECC (30 and 60 min). **(B)** Time course of lymphocytes (left y-axis) and monocytes (right y-axis) over the same interval. **(C)** Endpoint comparisons among Control, Sham, and ECC for WBC and neutrophils. **(D)** Endpoint comparisons for lymphocytes and monocytes.

To assess the ability of this system to replicate the hematologic changes induced by ECC, we first conducted a complete blood cell analysis. Following the initiation of ECC in mice, we noted rapid changes in peripheral leukocyte populations. During the initial 60 min, total white blood cell counts (WBC) increased in a time-dependent manner, primarily due to a concurrent rise in neutrophils, which exhibited significant pairwise differences between time points (Figure 3A). Conversely, lymphocyte numbers decreased during this period, while monocytes demonstrated a modest yet consistent increase (Figure 3B). Comparisons among endpoint groups confirmed these trends: relative to sham and non-instrumented controls, ECC mice displayed pronounced leukocytosis and neutrophilia, alongside reduced lymphocyte counts and elevated monocyte levels (Figures 3C,D).

Collectively, these data suggest that ECC triggers an early, innate-skewed hematologic response characterized by rapid neutrophil mobilization and relative lymphopenia, which becomes apparent within the first hour following circuit initiation. This significant leukocyte remodeling reflects the acute inflammatory cascade observed in clinical ECC scenarios, where neutrophil activation and lymphoid suppression contribute to postoperative complications, including systemic inflammatory response syndrome (SIRS) (Misokalou et al., 2025). The pronounced increase in neutrophils, alongside the decrease in lymphocytes and moderate elevation of monocytes, indicates that our murine ECC model accurately replicates the hallmark hematologic and inflammatory changes associated with clinical ECC.

### 3.2 ECC induced early thrombocytopenia with concurrent changes in platelet size indices

**FIGURE 4:**
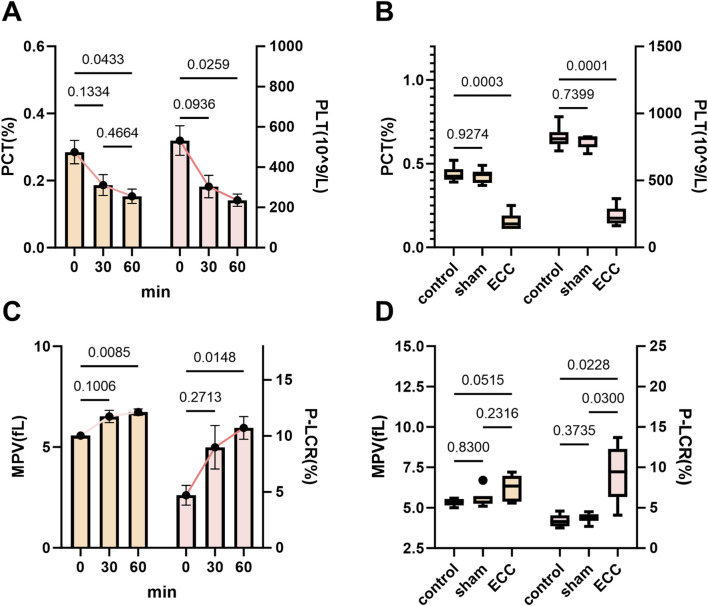
ECC induced early thrombocytopenia with concurrent changes in platelet size indices. **(A)** Time course of PCT (left y-axis) and PLT (right y-axis) at 0, 30, and 60 min after ECC initiation. **(B)** Endpoint comparisons among Control, Sham, and ECC for PCT and PLT. **(C)** Time course of MPV (left) and P-LCR (right) over 0–60 min. **(D)** Endpoint comparisons for MPV and P-LCR.

Building on the characterization of ECC-induced inflammatory leukocyte dynamics, we subsequently examined the hemostatic changes occurring during early ECC. Within 60 min of initiating ECC, both plateletcrit (PCT) and platelet count (PLT) exhibited significant declines (Figure 4A), with endpoint analyzes confirming marked thrombocytopenia in the ECC group compared to sham and non-instrumented controls (Figure 4B). This swift platelet loss reflects the clinical context of ECC-associated thrombocytopenia, where artificial surfaces and shear stress promote platelet activation and sequestration. In contrast, mean platelet volume (MPV) and the platelet–large cell ratio (P-LCR) increased during early ECC (Figure 4C), suggesting a transition toward larger, more reactive platelets. Furthermore, terminal assessments revealed a significant increase in P-LCR among ECC animals (Figure 4D), indicating an increased proportion of larger circulating platelets at the endpoint.

This inverse relationship—thrombocytopenia associated with macrothrombocytosis—is consistent with a state of high platelet turnover, indicating ongoing platelet activation on the surface of the artificial circuit and compensatory hematopoietic activity (Wang et al., 2021). However, based on the current data, it is not possible to definitively distinguish whether the observed platelet phenotype primarily reflects peripheral platelet consumption, enhanced platelet clearance, or activation of compensatory thrombopoietic pathways. These alterations create a prothrombotic and proinflammatory environment that predisposes to end-organ injury, as activated platelets not only facilitate thrombus formation but also enhance the inflammatory cascade through cytokine release and leukocyte interaction (Tucker et al., 2021).

Accordingly, future studies will incorporate direct markers of thrombopoiesis and platelet production, including analysis of the immature platelet fraction (IPF), measurement of circulating thrombopoietin (TPO) levels, and, where feasible, bone marrow–based assessments of megakaryopoiesis. Integration of these complementary readouts will allow direct discrimination between peripheral platelet consumption and true compensatory thrombopoietic responses, thereby providing a more mechanistically grounded interpretation of platelet dysregulation during extracorporeal circulation.

### 3.3 ECC induced metabolic acidosis with compensated systemic homeostasis

**FIGURE 5:**
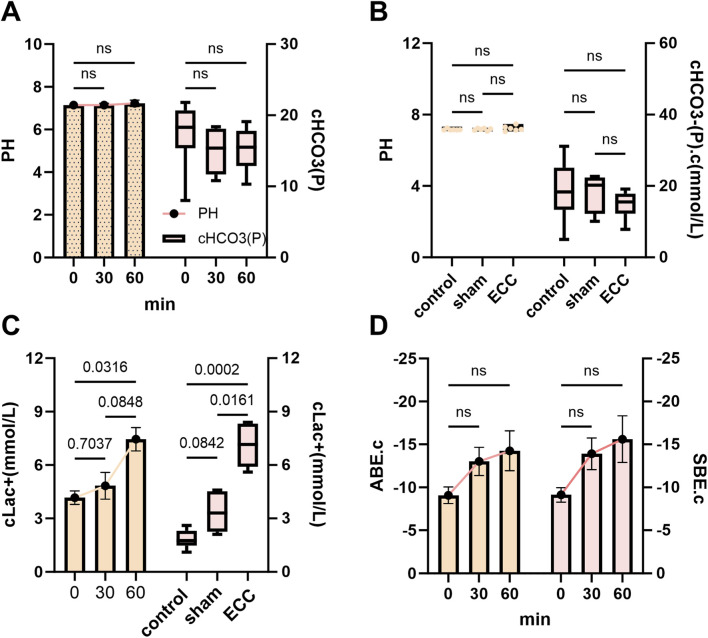
Metabolic acidosis with compensated systemic homeostasis. **(A)** Time courses of pH (left y-axis) and plasma bicarbonate cHCO_3_^-^(P) (right y-axis) measured at 0, 30, and 60 min after ECC initiation. **(B)** Endpoint comparisons among Control, Sham, and ECC for pH and cHCO_3_^-^(P). **(C)** Time course (left) and endpoint group comparison (right) of blood cLac^+^. **(D)** Time courses of actual and standard base excess (ABE.c, SBE.c). ns, not significant.

Building on the insights regarding ECC-induced hematologic and hemostatic alterations, we further examined the acid-base and metabolic profiles to elucidate the systemic metabolic stress experienced during early ECC. ECC induced a critical state of compensated metabolic stress, characterized by tissue hypoperfusion and systemic inflammation that promoted anaerobic metabolism, while physiological buffers preserved acid-base homeostasis (Jentzer et al., 2022). During the initial hour of ECC, blood lactate (cLac^+^) progressively increased (Figure 5C, left), indicating a significant oxygen debt at the tissue level, which is a hallmark of impaired tissue perfusion and mitochondrial dysfunction. Nevertheless, this metabolic burden was effectively mitigated by integrated compensatory mechanisms: arterial pH and plasma bicarbonate (cHCO_3_^-^(P)) remained stable across all time points (Figure 5A), with no significant differences observed among groups at the endpoint (Figure 5B). The consistently negative yet stable values of actual and standard base excess (ABE.c and SBE.c) throughout the procedure (Figure 5D) further underscored a state of fully compensated metabolic acidosis.

This dissociation between rising lactate and preserved pH illustrates a precarious equilibrium—early lactate accumulation signals underlying hypoperfusion and mitochondrial dysfunction, while the intact acid-base parameters demonstrate the remarkable capacity of systemic buffers (including bicarbonate and hemoglobin) to mitigate overt acidemia during the initial phase of ECC (Jentzer et al., 2022). The elevated endpoint lactate in the ECC group compared to controls (Figure 5C, right) indicates the presence of early metabolic stress during short-term ECC, preceding overt acid–base imbalance or hemodynamic instability. Collectively, these data demonstrate that our murine ECC model faithfully recapitulates the early metabolic signature of clinical ECC, characterized by compensated metabolic acidosis driven by tissue hypoperfusion.

### 3.4 ECC induced electrolyte imbalance and cardiac vulnerability

**FIGURE 6:**
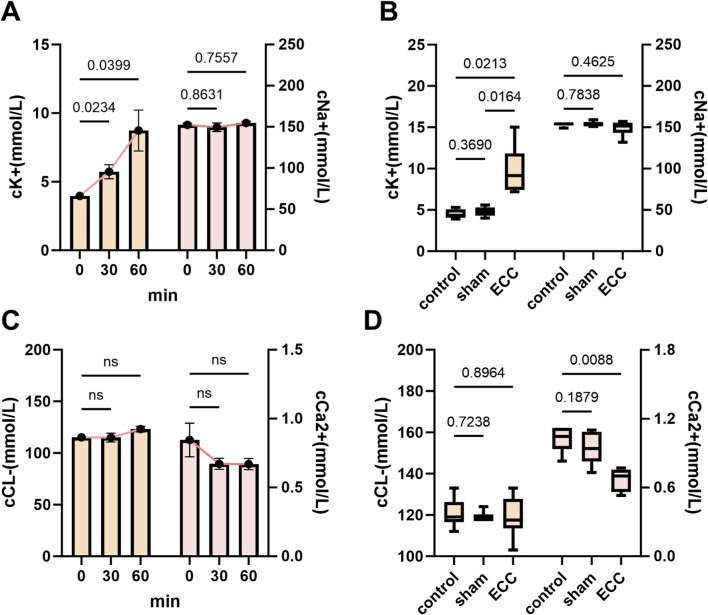
ECC-induced electrolyte imbalance and cardiac vulnerability. **(A)** Time courses of serum cK^+^ (left y-axis) and cNa^+^ (right y-axis) measured at 0, 30, and 60 min after ECC initiation. **(B)** Endpoint comparisons among Control, Sham, and ECC groups for cK^+^ and cNa^+^. **(C)** Time courses of serum cCl^−^ (left y-axis) and cCa^2+^ (right y-axis) measured at 0, 30, and 60 min after ECC initiation. **(D)** Endpoint comparisons among Control, Sham, and ECC groups for cCl^−^ and cCa^2+^. ns, not significant.

Building on the characterization of inflammatory, hemostatic, and metabolic responses, we further examined electrolyte and hematologic dynamics to elucidate the systemic homeostatic breakdown during ECC—providing key evidence that validates the clinical relevance of our model. Serum potassium (cK^+^) in the ECC group demonstrated a significant time-dependent increase (Figure 6A), with endpoint levels markedly higher than those observed in the control and sham groups (Figure 6B), indicating progressive hyperkalemia. This finding has profound physiological implications: elevated extracellular potassium disrupts cardiac membrane potential, impeding action potential propagation and heightening the risk of arrhythmias—characteristics of clinical ECC-associated cardiac electrical instability. Such elevations in extracellular potassium can depolarize cardiomyocyte resting membrane potential, slow impulse conduction, and increase arrhythmogenic susceptibility, consistent with cardiac electrical instability observed in clinical extracorporeal support settings. In contrast, serum sodium (cNa^+^) remained stable across groups and time points (Figures 6A,B), arguing against generalized hemodilution as the primary driver and supporting a potassium-predominant disturbance.

Time-course analysis of chloride (cCl^−^) and ionized calcium (cCa^2+^) showed a mild upward trend in cCl^−^ and a slight downward trend in cCa^2+^ within the ECC group, neither reaching statistical significance (Figure 6C). At the endpoint, cCl^−^ remained modestly elevated without significance, whereas cCa^2+^ in the ECC group was significantly lower than in controls (P < 0.05) and closely aligned with values observed in the sham group (Figure 6D). Ionized calcium is essential for excitation–contraction coupling via troponin C activation and sarcomeric cross-bridge formation; thus, reduced cCa^2+^ may contribute to impaired myocardial contractility and heightened cardiac vulnerability during ECC (Jeong and Lim, 2020). Together, the coexistence of hyperkalemia and reduced ionized calcium represents a combined electrophysiological and contractile stressor that may predispose to arrhythmia and pump dysfunction during extracorporeal support.

Overall, the electrolyte profile observed here—pronounced hyperkalemia with concomitant reduction in ionized calcium in the setting of stable sodium—supports a pattern of selective homeostatic disruption during ECC.

### 3.5 ECC induced preserved hematologic indices and limited hemodilution

Given that hemodilution is a recognized potential confounder in ECC studies, particularly in small-animal models, we assessed whether dilutional effects occurred during ECC and whether they could influence the interpretation of hematologic, metabolic, and electrolyte data.

**FIGURE 7:**
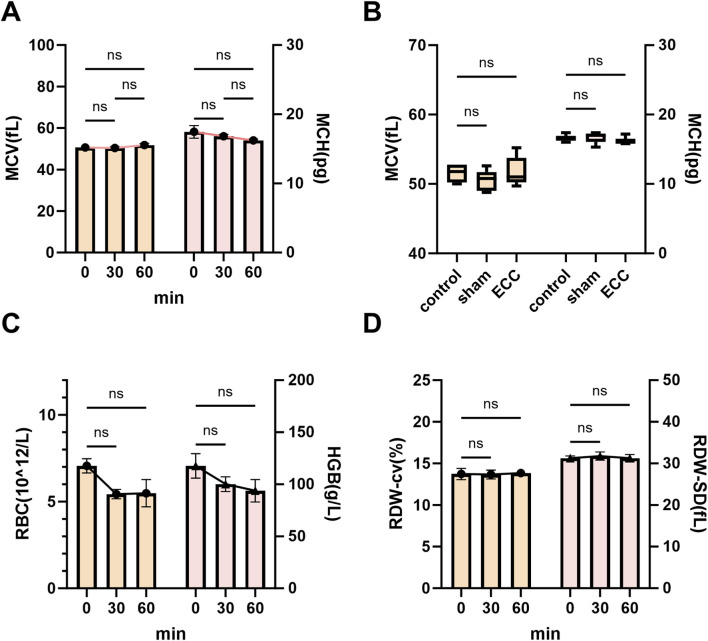
ECC induced preserved hematologic indices and limited hemodilution. **(A)** Time courses of MCV (left y-axis) and MCH (right y-axis) at 0, 30, and 60 min after ECC initiation. **(B)** Endpoint comparisons among Control, Sham, and ECC groups for MCV and MCH. **(C)** Time courses of RBC count (left y-axis) and HGB (right y-axis) at 0, 30, and 60 min after ECC initiation. **(D)** Endpoint comparisons among Control, Sham, and ECC groups for RBC count and HGB. ns, not significant.

Dilution-sensitive hematologic parameters were analyzed at baseline (0 min), 30 min, and 60 min following ECC initiation, including red blood cell (RBC) count, hemoglobin (Hgb) concentration, red cell distribution width (RDW-CV and RDW-SD), mean corpuscular volume (MCV), and mean corpuscular hemoglobin (MCH) (Figure 7). Throughout the 60-min ECC period, RBC counts and hemoglobin levels remained stable, with no significant temporal changes or differences compared with sham-operated or non-instrumented control groups (Figure 7C), indicating the absence of overt dilutional anemia. RDW-CV and RDW-SD values also remained unchanged (Figure 7D), suggesting preserved erythrocyte size homogeneity and arguing against substantial hemolysis, fluid overload, or pathological red blood cell turnover. Similarly, MCV and MCH showed no significant temporal or intergroup differences (Figures 7A,B), supporting the absence of meaningful erythrocyte swelling, shrinkage, or intracellular hemoglobin dilution. Collectively, these findings indicate that erythrocyte count, size distribution, and hemoglobin content remained stable during short-term ECC.

Taken together, these hematologic data are consistent with the experimental design features intended to limit dilutional effects, including the short ECC duration (60 min) and restricted blood sampling volumes. Under the present conditions, systemic hemodilution was effectively controlled and did not represent a dominant contributor to the observed metabolic or electrolyte alterations, such as lactate accumulation or electrolyte imbalance described above.

Nevertheless, hemodilution remains an inherent limitation of the current model. To further reduce blood loss and improve animal welfare in future studies, we plan to adopt advanced microsampling and analytical approaches, such as micro-volume blood gas analyzers and multiplex assays requiring minimal sample volumes. These strategies will allow precise physiological and biochemical assessment while substantially reducing per-sample blood volume, thereby enhancing data fidelity and animal welfare.

### 3.6 ECC induced early systemic inflammatory responses and organ stress–associated biomarker changes

**FIGURE 8:**
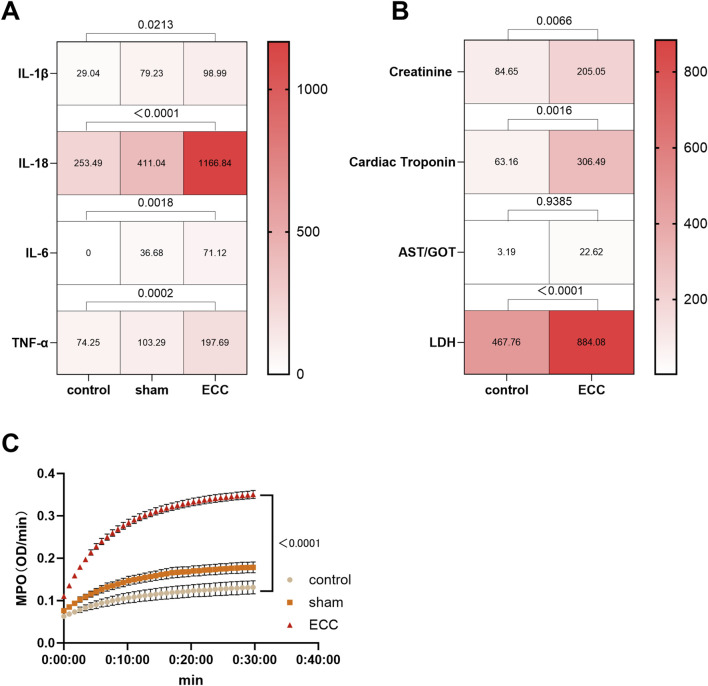
ECC induced early systemic inflammatory responses and organ stress–associated biomarker changes. **(A)** Heatmap representation of plasma pro-inflammatory cytokine levels, including IL-1β, IL-18, IL-6, and TNF-α, measured in Control, Sham, and ECC groups at the end of the 60-min experimental period. Color intensity reflects relative concentration levels. Statistical comparisons among groups are indicated above the heatmap. **(B)** Heatmap comparison of plasma organ stress–associated biomarkers between Control and ECC groups at 60 min, including creatinine, cardiac troponin, AST/GOT, and LDH. Color intensity represents relative biomarker levels. P values for group comparisons are shown above each parameter. **(C)** Time-course analysis of myeloperoxidase (MPO) activity over a 30-min reaction period in plasma samples from Control, Sham, and ECC groups. MPO activity is expressed as optical density per minute (OD/min). ns, not significant.

To further characterize the early systemic responses elicited by short-term ECC, we next examined circulating inflammatory mediators and organ injury–associated biochemical markers at the 60-min ECC time point (Figure 8).

As shown in (Figure 8A), ECC induced a robust early inflammatory response, reflected by significantly elevated plasma levels of pro-inflammatory cytokines, including interleukin-1β (IL-1β), interleukin-6 (IL-6), tumor necrosis factor-α (TNF-α), and interleukin-18 (IL-18), compared with both sham-operated and non-instrumented control groups. These findings indicate rapid activation of innate immune signaling pathways following blood exposure to artificial circuit surfaces during ECC.

In parallel, we assessed a panel of plasma biomarkers associated with early organ stress or injury across multiple organ systems (Figure 8B). Renal stress was evidenced by a significant increase in plasma creatinine levels in the ECC group. Cardiac involvement was reflected by elevated circulating cardiac troponin and lactate dehydrogenase (LDH), indicating early myocardial cellular stress and membrane permeability alterations. Hepatic stress was suggested by increased plasma aspartate aminotransferase (AST/GOT) activity, while pulmonary involvement was indicated by a significant elevation in myeloperoxidase (MPO) activity, consistent with early neutrophil activation and pulmonary inflammatory signaling.

Importantly, these biochemical alterations were detected within a short, 60-min ECC exposure window, and therefore represent early inflammatory activation and organ injury–associated signals rather than established or irreversible organ dysfunction. Accordingly, the observed biomarker changes should be interpreted as early indicators of organ stress and inflammatory engagement, consistent with the acute phase of ECC exposure.

Collectively, these results demonstrate that short-term ECC in this murine model is sufficient to trigger rapid systemic inflammation accompanied by measurable biochemical signals associated with early organ stress (kidney, heart, liver, and lung). These findings provide biochemical evidence of early systemic and organ-specific responses to ECC, distinct from definitive multiorgan failure or terminal organ injury.

## 4 Discussion

The present study establishes a miniaturized murine extracorporeal circulation model for the characterization of early systemic hematologic, metabolic, and electrolyte dynamics during short-term extracorporeal circulation (ECC) exposure. Previous reports suggested that blood–artificial surface interaction can induce early innate immune activation following (Wiener-Kronish and Nozari, 2014), platelet homeostasis altered during ECC (Li et al., 2024; Jung et al., 2025), arterial pH and bicarbonate remained stable during early ECC exposure (Ma et al., 2017), and early disruptions in systemic electrolyte regulation (Van den Eynde et al., 2021). Within a 60-min ECC window, we observed coordinated alterations across multiple physiological domains, including hematologic, metabolic, electrolyte, and inflammatory parameters. Specifically, ECC induced rapid changes in leukocyte distributions, characterized by neutrophilia, lymphopenia, and monocytosis, Platelet count and plateletcrit declined, accompanied by increases in platelet size indices. Metabolically, circulating lactate levels increased progressively. In parallel, selective electrolyte perturbations were observed, including time-dependent hyperkalemia, stable sodium levels, and modest alterations in chloride and ionized calcium. Importantly, these observed changes represent early systemic perturbations during ECC initiation, rather than definitive evidence of tissue hypoperfusion, shock, or irreversible organ dysfunction. The stability of core physiological parameters, including body temperature and acid–base balance, underscores the controlled and reproducible nature of the experimental platform. Collectively, this model provides a robust framework for investigating the initial biological responses to ECC, offering mechanistic insight into how hematologic, metabolic, electrolyte, and inflammatory processes begin to diverge shortly after ECC initiation.

Compared to existing murine ECC models (Mukhopadhyayet al., 2018; Natanov et al., 2019; Smood et al., 2025), our system offers superior reproducibility and accessibility through its pre-configured, single-use circuit design, which minimizes priming volume and operational complexity. This advantage is critical for large-scale therapeutic screening and genetic studies. The observed “inflammatory storm,” a fundamental aspect of post-ECC complications, is primarily triggered by the interaction of blood with the artificial surfaces of the circuit (Dhar et al., 2019). This interaction activates complement cascades, platelets, and endothelial cells. The substantial increase in neutrophils, which are the primary effector cells of innate immunity, directly results from this activation and plays a critical role in subsequent end-organ damage through the release of proteases and reactive oxygen species (Yang et al., 2024). Platelet-derived mediators (e.g., PF4, CD40L) may further amplify neutrophil recruitment and activation, creating a positive feedback loop that exacerbates inflammation (Ribeiro et al., 2019). These findings also were supported in our model. Notably, because our inflammatory readouts were limited to IL-1β, IL-6, TNF-α, and IL-18, the broader inflammatory landscape (e.g., complement activation and additional cytokine/chemokine networks) remains incompletely characterized and should be addressed in future studies.

A pivotal finding of our study is the development of significant metabolic acidosis, driven by lactate accumulation (Soliman et al., 2016). This condition indicates a shift to anaerobic metabolism, likely resulting from multiple factors, including hemodilution-induced anemia, microemboli, and dysregulated inflammatory signaling that disrupts microcirculatory flow, rather than global circulatory failure (Mallat and Vallet, 2021). The resulting electrolyte imbalances hold paramount clinical significance. Hyperkalemia, a life-threatening complication, can be attributed to both acidosis-induced transcellular shifts of potassium and potential impairment of renal perfusion (Malik, 2012). The notable hypocalcemia, often overlooked in experimental models, can directly impair cardiac contractility and exacerbate coagulopathy, reflecting critical challenges in managing post-cardiotomy patients (Srichuachom et al., 2025).

Our miniaturized murine ECC model represents a significant advancement over previous systems (Natanov et al., 2019; Kharnaf et al., 2022; Madrahimov et al., 2023). The seminal work by Haverich et al. (2017) successfully established a murine CPB model to address the specific challenges of deep hypothermic circulatory arrest. Building directly upon this foundational methodology, we developed a standardized, accessible, and cost-effective circuit kit. This technical advancement substantially lowers the barrier to entry, enabling a shift in research focus toward sustained investigations of ECC pathophysiology under normothermic conditions. Importantly, the present model is designed to investigate early initiation-phase biology under controlled conditions, rather than to replicate prolonged extracorporeal life support or to infer late-stage organ failure trajectories.

## 5 Limitations

Despite these advancements, we acknowledge specific limitations inherent in the current model setup. First, the circuit employs a conventional membrane oxygenator with a static gas supply, which lacks the dynamic responsiveness required to accommodate rapid physiological fluctuations in mice and may constrain fine control of gas exchange. This limitation may compromise gas exchange efficiency and safety. In addition, the present platform uses a roller-type peristaltic pump, which may warrant consideration with respect to potential air entrainment and drainage-related instability when compared with centrifugal pump systems in miniaturized circuits. Future iterations will explore the feasibility of integrating a centrifugal pump to further improve operational safety and circuit stability. Moreover, the invasive nature of multi-site cannulation and monitoring, including the use of a rectal probe for core temperature measurement, introduces further procedural stress. Looking ahead, our ongoing efforts aim to refine technology to minimize invasiveness and enhance data fidelity. To address the limitations of gas supply control, future iterations will incorporate an adaptive gas supply system, as pioneered by Wang et al. (2025). Second, direct hemodynamic measurements were not obtained. Continuous monitoring of mean arterial pressure (MAP), heart rate trends, cardiac output, and microcirculatory perfusion was not performed, limiting mechanistic interpretation of systemic perfusion. Consequently, conclusions regarding systemic hypoperfusion, microcirculatory failure, or shock-like states cannot be made, and metabolic changes should be interpreted as indicators of early systemic stress rather than confirmed perfusion deficits. Future work will prioritize integration of invasive arterial pressure monitoring and microcirculatory imaging to strengthen physiological inference. Third, although priming volume and blood sampling were minimized, residual hemodilution cannot be fully excluded in this small-animal ECC model and may subtly influence sensitive metabolic and electrolyte measurements. To further mitigate this confounder and improve animal welfare, future studies will adopt microsampling strategies and low-volume analytical platforms to reduce blood loss while preserving analytical precision. In addition, the observation window was limited to 60 min, which restricts interpretation to acute initiation-phase responses; delayed inflammatory sequelae, survival outcomes, and progressive organ dysfunction cannot be assessed in the current study. Accordingly, while we included early organ stress–associated plasma biomarkers, these readouts do not establish structural tissue injury in the absence of histological confirmation or longitudinal functional outcome assessment. Fourth, the sample size was modest (n = 6 per group), and no formal *a priori* power calculation was performed, consistent with an initial feasibility- and model-characterization design. Larger, outcome-driven cohorts will be required to confirm effect sizes across multiple endpoints, strengthen statistical power, and improve generalizability. Fifth, this study was conducted exclusively in male C57BL/6 mice, which may limit the generalizability of the findings. Biological sex is known to influence inflammatory responses, platelet function, and metabolic regulation—processes central to ECC-associated pathophysiology. While the use of male mice reduced variability related to estrous cycling during initial model establishment, future studies will incorporate female animals to evaluate sex-dependent responses and enhance translational relevance.

Collectively, these limitations indicate that the current platform is optimized for studying early, acute-phase systemic responses to short-term ECC exposure, rather than for definitive assessment of hemodynamic failure or delayed organ dysfunction.

## 6 Conclusion

In summary, this study establishes a miniaturized murine ECC model to characterize early, acute-phase systemic responses during short-term ECC. Rather than recapitulating advanced organ failure, the model provides a reproducible framework for investigating initial hematologic, metabolic, electrolyte, and inflammatory perturbations, and for preclinical evaluation of strategies targeting early ECC-associated stress.

## Data Availability

The original contributions presented in the study are included in the article/supplementary material, further inquiries can be directed to the corresponding authors.
